# Fault Diagnosis Method for Imbalanced Data Based on Multi-Signal Fusion and Improved Deep Convolution Generative Adversarial Network

**DOI:** 10.3390/s23052542

**Published:** 2023-02-24

**Authors:** Congying Deng, Zihao Deng, Sheng Lu, Mingge He, Jianguo Miao, Ying Peng

**Affiliations:** 1 School of Advanced Manufacturing Engineering, Chongqing University of Posts and Telecommunications, Chongqing 400065, China; 2 CNPC Chuanqing Drilling Engineering Co., Ltd., Chengdu 610051, China; 3 College of Automation, Chongqing University of Posts and Telecommunications, Chongqing 400065, China

**Keywords:** fault diagnosis, generative adversarial network, attention mechanism, feature fusion

## Abstract

The realization of accurate fault diagnosis is crucial to ensure the normal operation of machines. At present, an intelligent fault diagnosis method based on deep learning has been widely applied in mechanical areas due to its strong ability of feature extraction and accurate identification. However, it often depends on enough training samples. Generally, the model performance depends on sufficient training samples. However, the fault data are always insufficient in practical engineering as the mechanical equipment often works under normal conditions, resulting in imbalanced data. Deep learning-based models trained directly with the imbalanced data will greatly reduce the diagnosis accuracy. In this paper, a diagnosis method is proposed to address the imbalanced data problem and enhance the diagnosis accuracy. Firstly, signals from multiple sensors are processed by the wavelet transform to enhance data features, which are then squeezed and fused through pooling and splicing operations. Subsequently, improved adversarial networks are constructed to generate new samples for data augmentation. Finally, an improved residual network is constructed by introducing the convolutional block attention module for enhancing the diagnosis performance. The experiments containing two different types of bearing datasets are adopted to validate the effectiveness and superiority of the proposed method in single-class and multi-class data imbalance cases. The results show that the proposed method can generate high-quality synthetic samples and improve the diagnosis accuracy presenting great potential in imbalanced fault diagnosis.

## 1. Introduction

The rolling bearing is an important part of the rotating machinery system, whose failures may cause huge economic losses or even threats to people’s lives. Therefore, it is of great significance to develop an advanced and effective fault diagnosis method to monitor bearing failures. In recent years, many data-driven fault diagnosis methods have been proposed based on machine learning and promoted the development of intelligent fault diagnosis technology [1,2,3]. Among them, deep learning (DL) methods have attracted much attention as they can process mechanical signals quickly and effectively, which contributes to reliable fault diagnosis results [4,5].

Vibration signals contain abundant health information and have been commonly used for bearing fault diagnosis [6,7]. Compared with knowledge-based and traditional machine learning methods, DL-based methods can automatically extract features from measured signals and achieve more accurate diagnoses. The convolutional neural network (CNN) is one of the commonly used DL-based methods for fault diagnosis. Chen et al. [8] constructed a CNN model for fault classification based on the time-frequency feature of vibration signals. Wang et al. [9] proposed a bottleneck layer-optimized CNN method for fault diagnosis based on rearranged data. Huang et al. [10] proposed a multi-scale cascade CNN to enhance the feature extract ability and diagnosis accuracy. Although CNN has made some progress in the field of fault diagnosis in recent years, there are still challenges.

The datasets for training CNN models are assumed abundant and balanced in the aforementioned works. However, the mechanical equipment operates in a normal state for a long-time in practical engineering, resulting in insufficient fault samples and imbalanced data occasions. The diagnosis model will sacrifice the accuracy of the minority class to obtain higher accuracy of the entire dataset facing imbalanced data, which greatly deteriorates the diagnosis accuracy [11]. Since under-sampling for the majority class samples will waste much information about the majority classes, methods focusing on the utilization of limited samples have been developed. The over-sampling method is a kind of data-based and effective method for the data augmentation of a minority class [12]. The synthetic minority over-sampling technique (SMOTE) is a typical over-sampling method [13], which has been adopted for data augmentation in fault diagnosis areas [14,15]. However, SMOTE generates new samples through interpolation in the existing samples, which will easily lead to over-fitting.

To address the above challenge, a generative adversarial network (GAN), which focuses on learning the distribution of the original real samples through an adversarial process, is adopted to solve the data imbalance problem. Once the GAN is well trained, its generator can generate samples with similar distribution to the real samples [16]. GAN has been widely applied to many fields, including medical image generation, image enhancement, neural style transfer, etc. [17,18]. GAN has also been introduced into the mechanical fault diagnosis field to deal with limited data problems. Wang et al. [19] constructed a deep convolution GAN (DCGAN) to generate new samples for minority class samples. Compared with raw signals, samples with enhanced features, such as features extracted from unsupervised learning [20], frequency spectrum [21,22], and time-frequency images [23,24,25] can improve the learning ability of GAN. However, GAN always faces the problem of unstable training, resulting in poor sample generations. Therefore, it is necessary to ensure the stability of the training process to generate more realistic samples, so as to improve the accuracy of fault diagnosis.

To address the above challenge, a fault diagnosis method for imbalanced is proposed aiming at improving the diagnosis accuracy from three aspects, which are multi-signal fusion, augmentation of high-quality data based on improved DCGAN (IDCGAN), and improved classifier The main contributions of this paper are summarized as follows:

1. A data augmentation method based on IDCGAN is proposed to solve the imbalanced data problem. The self-attention mechanism is introduced in DCGAN to improve the generated sample quality, and the spectral normalization (SN), two time-scale update rules (TTUR), and Wasserstein distance and gradient penalty are applied for stabilizing the training process of DCGAN.
2. A multi-signal fusion method is proposed to mitigate the effects of imbalanced data and improve the diagnosis performance. The synchronizing vibration signals measured by multiple sensors are separately transformed into 2-D time-frequency images, which are fused through the pooling and splicing operations to provide more fault features.
3. An enhanced classifier based on residual networks is proposed to achieve higher diagnostic accuracy. The Convolutional Block Attention Module (CBAM) is introduced in the pre-trained residual network for better capturing the important space and channel features.

The remainder of the paper is organized as follows. In Section 2, the basic theories are given. In Section 3, the details of the proposed diagnosis framework are provided. In Section 4, The experimental studies based on two different rotating components are provided to validate the performance of the proposed method. Finally, the conclusions are presented.

## 2. Theoretical Backgrounds

### 2.1. Continuous Wavelet Transform

Compared with raw vibration signals, time-frequency images can provide more powerful state information [26], which contributes to better recognition results. CWT can provide the continuous translation and scaling of the wavelet basis function to obtain a class of wavelet family and transform time-series signals into other feature spaces. Therefore, CWT has been widely used for obtaining time-frequency images, whose definition is as follows:

(1) $$W_{f}\left(a,b\right)=\langle x\left(t\right),\psi_{a,b}\left(t\right)\rangle =\frac{1}{\sqrt{a}}\int_{-\infty }^{+\infty }x\left(t\right)\psi^{*}\left(\frac{t-b}{a}\right)dt,$$

where *a* represents the scaling factor, *b* is the translation factor, *x*(*t*) represents the input signals, *Ψ_a,b_*(*t*) is the wavelet basis function (WBF), Ψ* represents the complex conjugate function of *Ψ_a,b_*(*t*), and 〈⋅〉 represents the internal product.

WBF plays an important role in the wavelet transform and different WBF analyses have different results under the same signal. Compared with some popular WBFs, such as Db10, Coif5, and Meyer, the Morlet WBF has fine regularity, a high similarity coefficient, and a definite analytical equation, which is more suitable for the application of vibration signals [24]. Therefore, the Morlet WBF is adopted in this paper.

### 2.2. Generative Adversarial Network

**Figure 1:**
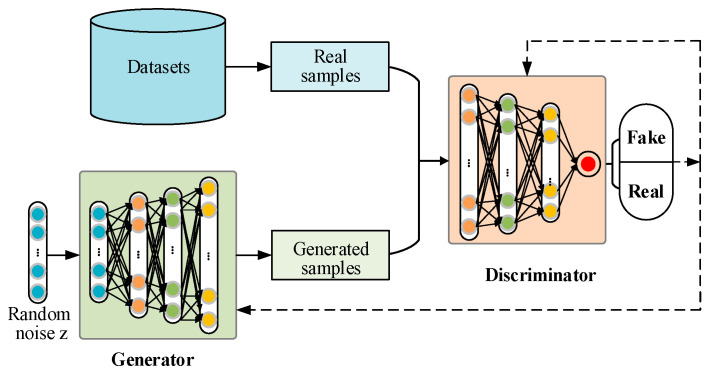
The basic structure of standard GAN.

As shown in Figure 1, a standard GAN consists of two basic networks: a generator (*G*) and a discriminator (*D*). The G makes use of random noise to generate fake samples, which are sent to *D* for identification together with real samples. The *D* needs to distinguish whether the input sample is real or fake as far as possible. These two networks are trained alternately and compete with each other, presenting adversarial learning. The goal of this training is to achieve the Nash equilibrium and the *G* can generate samples with the same distribution as real samples. The optimization of GAN is to minimize the generator loss and maximize the discriminator loss. That is, the predicted value from *D* for both the real sample and the generated sample is 0.5. The training process can be described as follows:

(2) $$\underset{G}{\min}\underset{D}{\max V}(D,G)=E_{x\sim Pdata(x)}[log(D(x))]+E_{z\sim Pz(z)}[log(1-D(G(z)))],$$

where *P_data_* is the distribution of real samples, *Pz* represents the prior distribution of the input noise, *G*(*z*) is the generated sample by the generator, and *D*(·) represents the predicted value from the discriminator.

DCGAN is a variant of GAN, which adopts the convolution and transposed convolution layers to replace the initial fully connected layers in the original GAN. Besides, the ReLU is adopted as the activation function of the G network and the Leaky ReLU is used as the activation function of the *D* network. DCGAN has been widely used on many occasions due to its improved training stability and the high quality of the generated samples.

### 2.3. Wasserstein Distance and Gradient Penalty

Wasserstein distance is used to measure the distance between two probability distributions [27]. Its definition can be illustrated as follows:

(3) $$W(P_{r},P_{g})=\underset{\gamma \sim \prod (P_{r},P_{g})}{\inf}E_{(x,y)\sim \gamma }\left[\left\|x-y\right\|\right],$$

where *P_g_* and *P_r_* represent the distribution of the generated sample and the raw sample, respectively. *γ* denotes an arbitrary joint distribution in the set of all joint distribution Π(*P_r_*, *P_g_*), *E_(x,y)_* means the expected value of the distance between x and y, and inf represents the infimum.

Wasserstein distance was introduced into GAN to increase the training stability [27]. However, Wasserstein GAN may have the problem of gradient explosion or gradient disappearance. Gradient penalty (*GP*) was introduced to improve the training stability of Wasserstein GAN and the quality of generated samples. The operation of *GP* can be expressed as follows:

(4) $$GP=\lambda E_{\hat{x}∼P}{}_{{}_{\hat{x}}}[{\left({\left\|\nabla_{\hat{x}}D(\hat{x})\right\|}_{2}-1\right)}^{2}],$$

where *λ* represents the gradient penalty coefficient, ${\left\|\cdot \right\|}_{2}$ is the L2-norm value of the gradient, $\hat{x}$ denotes the random sampling in the sample space containing real samples and generated samples.

### 2.4. Self-Attention Mechanism

The self-attention mechanism can effectively learn the non-local relationship of each important region in different samples [28,29]. In the self-attention mechanism, three 1 × 1 convolution layers with different output channels are applied to generate three different kinds of sub-feature maps (i.e., *f*(*x*), *g*(*x*), and *h*(*x*)) based on the input feature map *x*. Subsequently, the transposed matrix of sub-feature map *f*(*x*) is adopted to multiply *g*(*x*) to calculate corresponding attention weights, which are processed by the softmax activation function to obtain attention map *β_j,i_*. Then, the attention map and the output matrix *h*(*x*) are processed by the matrix multiplication to obtain the self-attention feature map, which is multiplied by a learnable coefficient α and added to the original input *x_j_* to obtain the final output feature map *y_j_*. The process of calculating self-attention maps can be expressed as follows:

(5) $$\beta_{j,i}=\frac{\exp(f{\left(x_{i}\right)}^{T}g(x_{j}))}{\sum_{i=1}^{N}\exp(f{\left(x_{i}\right)}^{T}g(x_{j}))},$$

(6) $$y_{j}=\alpha \sum_{i=1}^{N}\beta_{j,i}h(x_{i})+x_{j},$$

where *β_j_*_,*i*_ represents the attention score of the *i*-th pixel to the *j*-th pixel, *N* denotes the feature number of the input feature map

### 2.5. Convolutional Block Attention Module

**Figure 2:**
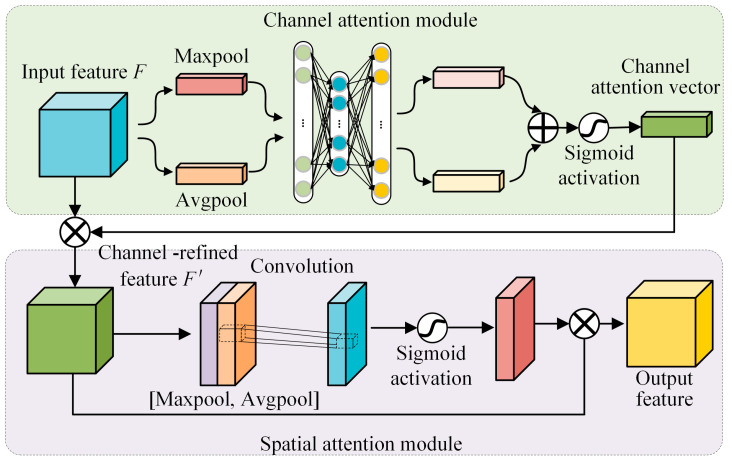
The basic structure of the CBAM.

CBAM can enhance the representation of specific regions and effectively improve the performance of CNN [30,31]. The basic structure of CBAM is illustrated in Figure 2, which mainly consists of two separate attention functions: channel attention and spatial attention. In the channel attention submodule, the input feature of each channel is firstly squeezed by global average-pooling and global max-pooling operations to obtain the characteristic feature of the whole spatial area. Then, the above features are input into the shared multi-layer perceptron (MLP) network to obtain further representational features. Finally, the two representation features from MLP are merged and activated to obtain the final channel attention vector. The process of channel attention can be expressed as follows:

(7) $$M_{output}(F)=\omega (MLP(AvgPool(F))+MLP(MaxPool(F))),$$

where *ω* represents the sigmoid activation function and *F* denotes the input feature map.

The structure of the spatial attention sub-module is shown in Figure 2, the channel refined feature is compressed through the global average-pooling and global max-pooling in the channel direction. Then, the two compressed spatial features are transformed into one channel through convolution operation. Finally, the spatial attention matrix is obtained after the sigmoid activation operation, which is multiplied by the channel refined feature to obtain the final output feature map. The process of the spatial attention module can be expressed as follows:

(8) $$M_{P}(F^{\prime })=\omega (f^{bxb}([AvgPool(F^{\prime });MaxPool(F^{\prime })])),$$

where *F*’ represents the channel-refined feature map, and *b* represents the size of the convolution kernel available in the convolution, which must be 3 or 7.

## 3. The Proposed Method

**Figure 3:**
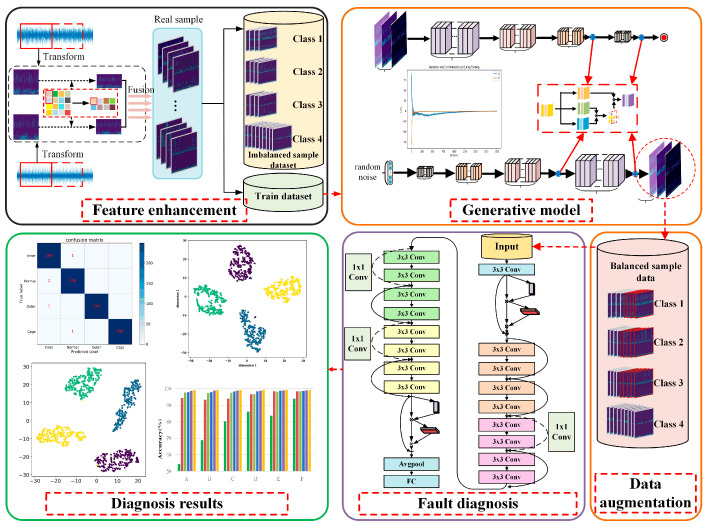
The framework of the proposed diagnosis method with imbalanced data.

To address the imbalanced data problem and improve the fault diagnosis performance, a novel bearing diagnosis method is proposed. The framework of the proposed method is shown in Figure 3, which mainly contains the following three steps:

Step 1: Feature enhancement with time-frequency feature and multiple sensor signals. The measured vibration signals from multiple sensors or directions are used to construct initial pairs of data samples. Then, CWT is adopted to process the samples to obtain the corresponding time-frequency information. Finally, pairs of time-frequency features are squeezed through the pooling operation and then fused by a splicing operation.

Step 2: Data augmentation based on a generative model. Improved DCGANs are constructed and trained to capture the distribution of real samples of different classes. Once the models are well-trained, they are used to generate new synthetic samples to augment the imperfect dataset.

Step 3: Fault diagnosis with enhanced ResNet. A DL-based classifier is constructed by introducing residual network and CBAM to improve the feature extraction ability of the model. Moreover, the expanded balanced dataset is used for classifier training, which aims to further improve the fault diagnosis accuracy.

### 3.1. Feature Enhancement and Feature Fusion

**Figure 4:**
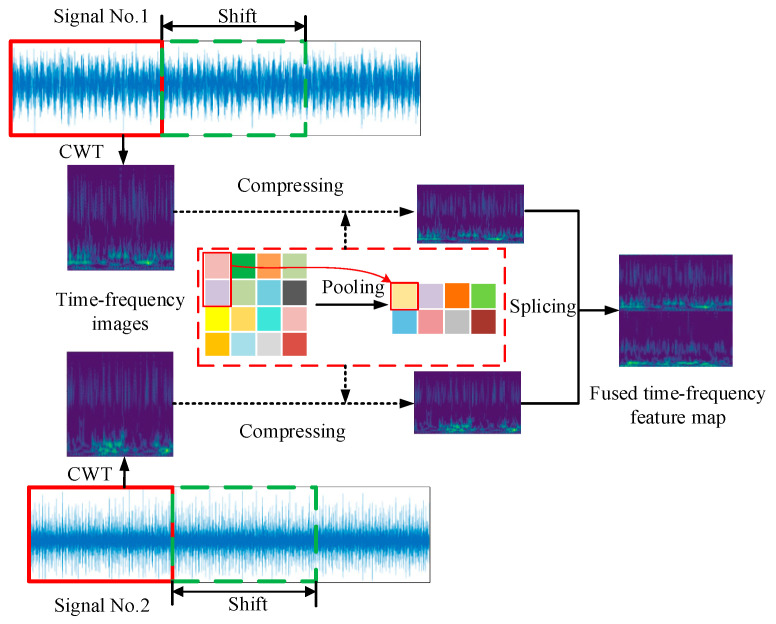
The flowchart of feature enhancement and feature fusion strategy.

Signals collected from different sensors or different directions can provide more health state information, which also benefits feature extraction and later classification. In addition, the time-frequency conversion of the signal through CWT can also provide more features compared with row time domain signals. Therefore, synchronizing signals collected from two different sensors are adopted to enhance the data feature in this study. The flowchart of feature enhancement and feature fusion strategy is shown in Figure 4. First, the synchronizing vibration signals are sliced into pairs of segments by sliding windows with fixed lengths. Subsequently, the pairs of signal segments are transformed by CWT to obtain corresponding time-frequency features, which are then squeezed through an average pooling operation with a size of 2 × 1. Finally, the squeezed features are merged by the up-down arrangement, and the image samples with fused time-frequency features are obtained.

### 3.2. Data Augmentation Based on Generative Model

Although DCGAN is beneficial for obtaining stable training, the unstable training problem still exists most of the time which will cause the mode collapse and gradient disappearance. In addition, SN is a novel weight normalization method that can achieve the Lipschitz constraint by constraining the L2 spectral norm of the weight matrix of each layer, and SN can enhance the training stability of GAN [32]. Therefore, an improved DCGAN (IDCGAN) is proposed by introducing the SN, Wasserstein distance with GP to enhance the training stability and generate satisfying samples [27]. The loss function of the proposed IDCGAN can be expressed as follows:

(9) $$Loss=\underset{y\sim p_{g}}{E}[D(y)]-\underset{x\sim p_{r}}{E}[D(x)]+\mu \underset{z\sim p_{z}}{E}[{\left(1-{\left\|H_{z}D(z)\right\|}_{2}\right)}^{2}],$$

(10) $${\left\|H_{z}D(z)\right\|}_{2}=\frac{\left|D(z_{1})-D(z_{2})\right|}{\left\|z_{1}-z_{2}\right\|},$$

where *D*(*x*) and *D*(*y*) represent the corresponding predicted value from the discriminator of the real sample and the generated sample, respectively, *μ* is a weight constraint coefficient, and *P_z_* denotes the joint distribution of the generated sample and the real sample. Once the Wasserstein distance and GP are introduced into DCGAN, the gradient tends to be near a certain stable value during the training process. Moreover, the Lipschitz constraint condition is further ensured by introducing the SN. These two strategies contribute to mitigating the vanishing gradient and exploding gradient problems.

**Figure 5:**
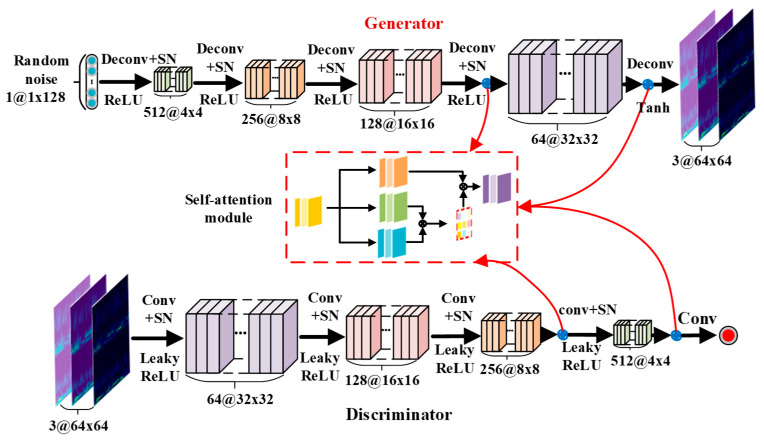
The basic structure of the improved DCGAN model.

Since the convolution kernel of the method using DCGAN is fixed, it cannot capture the global information. In contrast, the self-attention mechanism can extract features by establishing relationships between local and remote regions. Therefore, the performance of the generated model and the quality of the generated samples can be further improved by introducing the self-attention mechanism. The structure of the proposed IDCGAN is shown in Figure 5. SN is arranged after each convolutional layer and deconvolutional layer of both the discriminator and generator in place of the original batch normalization. Besides, the self-attention modules are added after the last two layers of both the generator and discriminator.

### 3.3. Fault Diagnosis Based on Enhanced ResNet

**Figure 6:**
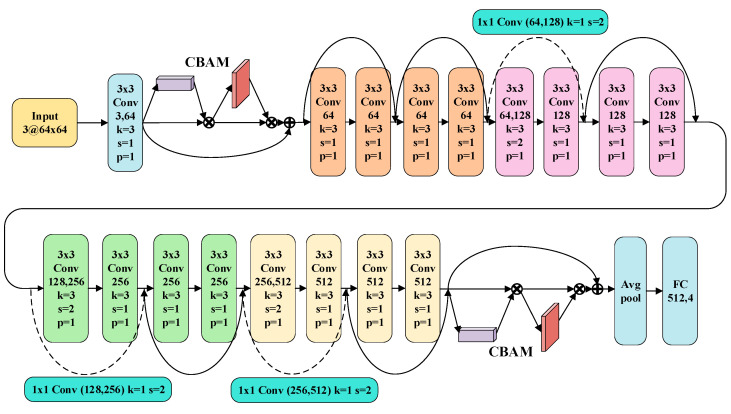
The basic structure of the proposed CBAM-ResNet.

Increasing the depth of the CNN can improve feature extraction ability. However, this operation can also lead to the disappearance of gradients [10]. The introduction of ResNet can solve this problem [33]. Therefore, ResNet is selected as the backbone of the classifier. Since the pre-trained model can help the network converge quickly in new conditions, partial structures of ResNet-18 with pre-train parameters are adopted as the initial classifier model in this paper. Besides, CBAM is introduced to improve the performance of ResNet-based diagnostic models (named CBAM-ResNet). The structure of the CBAM-ResNet is shown in Figure 6, and the CBAM is added after the first convolution layer and the last residual block.

## 4. Experimental Verification

In this section, two bearing datasets with different failure mechanisms, i.e., the full life cycle bearing failure dataset from Xi’an Jiaotong University and the Changxing Sumyoung Technology Co., Ltd. (Changzhou, China) (XJTU-SY) [34], and the artificial failure dataset from Case Western Reserve University (CWRU) [35] are selected to validate the effectiveness of the proposed method with imbalanced data.

### 4.1. Case 1: Bearing Dataset from XJTU-SY

#### 4.1.1. Dataset Description and Data Processing

**Figure 7:**
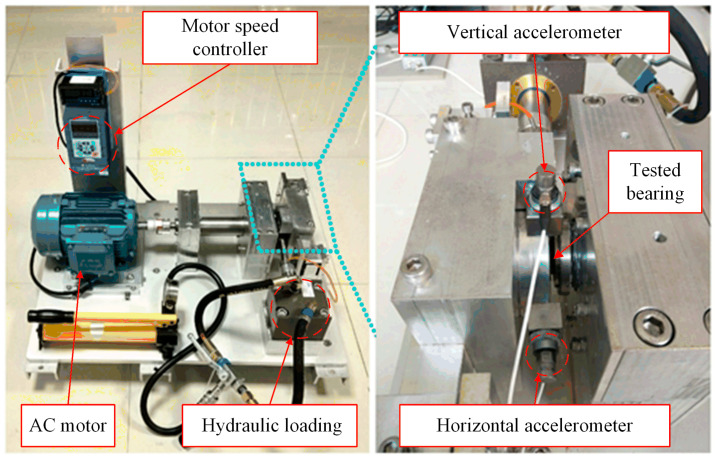
The test rig of the XJTU-SY.

The bearing dataset from XJTU-SY was collected from a bearing test rig which is shown in Figure 7. It mainly consists of the following parts: an AC motor, a hydraulic loading device, a tested bearing, a motor speed controller, and two accelerometers for measuring vertical and horizontal vibration [34]. The vibration signals were measured with a sampling frequency of 25.6 kHz. The dataset contains complete run-to-failure data of bearings which were acquired by conducting many accelerated degradation experiments. A total of 32,768 data points (i.e., 1.28 s) are recorded for each sampling until serious failure occurs.

**Figure 8:**
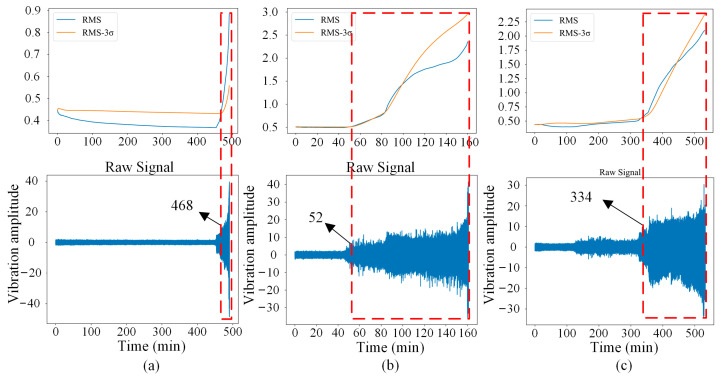
Signals and initial failure point of each fault. (**a**) IRF, (**b**) ORF, (**c**) BCF.

In this paper, the dataset collected under a rotational speed of 2250 rpm and a radial force of 11 kN is selected for experimental verification. Four different types of health states are considered for fault diagnosis, which are normal (Nor), inner race fault (IRF), outer race fault (ORF), and bearing cage fault (BCF). Since the dataset is a kind of full life cycle data, it is necessary to judge the initial occurred time of each fault from the whole data. Considering that the RMS-3σ rule is a smooth and more accurate fault identification method [36], the RMS-3σ rule is adopted to identify the early failure occurrence time point of each fault. The initial failure time of bearing 2–1, 2–2, and 2–3 (i.e., IRF, ORF, and BCF) are identified as 468, 52, and 334, respectively. That is, vibration signals after each initial failure point are used to construct data samples. The RMS-3σ results and corresponding initial failure points are shown in Figure 8.

It is well known that each sample should contain at least one circle sampling point. In this dataset, 683 sampling points can be collected per revolution according to the following equation:

(11) $$m=\frac{60fz}{n},$$

where *f*_z_ represents the sampling frequency and *n* represents the rotating speed.

Therefore, a sliding window with a constant length of 1280 points is adopted to construct samples. Subsequently, Z-score standard normalization is adopted to process the time series samples, which are then converted to time-frequency images with a size of 64 × 64 × 3 based on CWT and subsequent feature fusion. Finally, a total of 750 samples of each class are obtained, among which 250 samples are randomly selected to form the testing dataset. Moreover, a different number of minority samples are selected from the remaining samples of each class for IDCGAN model training according to the relative experiment settings. That is, if 500 samples are used as the majority sample, 100 minority samples are selected for IDCGAN model training with an imbalanced ratio of 0.2.

#### 4.1.2. Model Training and Sample Generation

In the training process of IDCGAN, the batch size and the iteration epoch are set as 32 and 2000 for each class, respectively. Besides, the discriminative ability of the discriminator is significantly stronger than the generative ability of the generator at the beginning; the TTUR strategy is adopted to balance the learning speed between the generator and the discriminator, which can improve the training stability. In this paper, the learning rates of the generator and the discriminator are 0.0001 and 0.0003, respectively.

**Figure 9:**
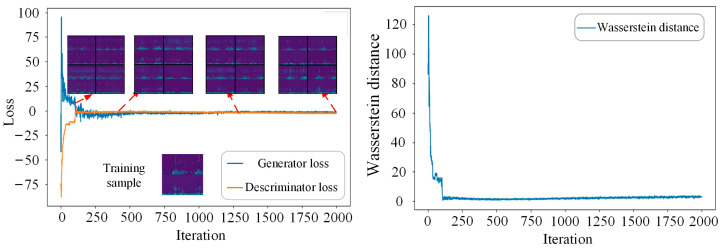
The training process of IDCGAN with 100 IRF samples.

The losses of the generator and discriminator, Wasserstein distance, together with visualization of the generated samples are adopted to better show the training process. Taking the IRF class with a limited sample number of 100 as an example, 100 real IRF samples are randomly selected from the training database as a minority class, which is used for training the IDCGAN model. The training process is shown in Figure 9. It can be seen that the discriminator loss, the generator loss, and the Wasserstein distance converge quickly and the quality of the generated samples becomes better as the iteration increases. The Nash equilibrium training is almost achieved when the iteration number increases to 1750, and the sample quality is very close to the real sample.

#### 4.1.3. Evaluation of the Generated Samples

To evaluate the quality of the generated samples, visualization results of the real samples and the generated samples are presented. Taking the limited sample number of 100 as an example, 100 real samples of each health state are adopted to train IDCGANs and then generate new synthetic samples.

**Figure 10:**
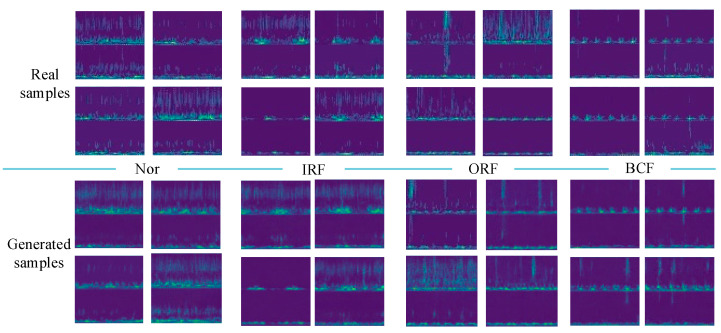
Comparison between the real samples and the generated samples.

The comparison results of the generated samples and corresponding real samples are shown in Figure 10. It is clear that all classes of the generated samples show favorable consistency in time-frequency features and also have some diversity, which means that the IDCGANs have captured the distribution of the initial training data and can generate high-quality synthetic samples.

To quantitatively evaluate the quality of the generated sample and show the superiority of the proposed feature fusion strategy, experiments in Table 1 are conducted based on the GAN-train strategy [37]. In all experiments mentioned above, IDCGAN is used for sample generation and CBAM-ResNet is used for fault classification. Time-frequency images of vibration signals from a single sensor are adopted to train the classifier in experiment A while the other experiments are based on fused time-frequency images from multiple sensors. For experiment B, real samples are used for fault identification and the generated samples in other experiments are obtained from the IDCGANs training with the same real samples of experiment B. According to reference [37], the diagnosis accuracies of experiments B and C are called GAN-base and GAN-train, respectively. Moreover, the distribution of synthetic samples is quite similar to the real samples once the values of the GAN-base and GAN-train are close. Experiments D and E are adopted to show the similarity and diversity between real and generated samples, which can be regarded as the extension of the GAN-train strategy.

As shown in Table 1, the diagnosis accuracy with information from single sensor (experiments A) is only 89.78% with a large standard deviation of 2.51%, which is increased to 96.89% with a smaller standard deviation of 1.21% by adopting the feature fusion method. This indicates that the proposed fusion method can provide more effective features which are beneficial for the classifier to learn more important information. By comparing experiments B and C, it can be seen that the GAN-train value with all generated samples is about 92.35%, which is close to the GAN-base value of 96.89% obtained based on real samples. In addition, both the diagnosis accuracy and standard deviation in experiment D are almost equal to the GAN-base value, which is obtained with half real samplesand half-generated samples of all classes. This further demonstrates the high similarity between the generated and real samples. In experiment E, the diagnosis accuracy is further improved to 98.83%, and the standard deviation is reduced to 0.61%, which proves the diversity of the samples generated by IDCGAN. From the above, it is reasonable to believe that the IDCGAN can be used to sample generation with similar diversity samples and for minority sample supplements.

#### 4.1.4. Single-Class Imbalanced Fault Diagnosis

Different evaluation criteria have been adopted to evaluate the similarity between the synthetic samples and the real samples. However, this operation will cause excessive similarity between the synthetic samples and the real samples, which ignores the sample diversity and will cause information redundancy [38]. Therefore, the generated samples are directly applied to expand the initially limited dataset. Moreover, the diagnosis accuracy can also be used as an indirect criterion to evaluate the generation ability of different generative models.

To verify the effectiveness of the proposed method in cases with limited fault samples, the single-class and multi-class imbalanced fault diagnosis experiments are established. Different initial fault samples are adopted to train the generative models, which are then used to generate new samples for data augmentation and achieve balanced data. Considering that the ORF occurs more frequently in all the full life cycle bearing experiments in the XJTU-SY dataset, ORF is selected as the minority class fault in single-class imbalanced experiments. It should be mentioned that the number of the majority class is 500, and the initial minority samples with different imbalance ratios are selected for training the generative models and expanding the minority class to 500 in the experiments, which are 0.5, 0.2 and 0.1 corresponding 250, 100 and 50 samples. To prove the superiority of the proposed method and different data augmentation methods, i.e., SMOTE, adaptive synthetic sampling (ADASYN), SN-assisted DCGAN (SN-DCGAN), and DCGAN based on Wasserstein distance with GP (WGANGP) are adopted for comparison. Meanwhile, different classifiers, i.e., CNN, ResNet, and CBAM-ResNet are used for comparative experiments to reduce the impact of the classifier on the results and verify the superiority of the improved classifier.

The CNN model is just a partial structure of the CBAM-ResNet without the skip connections and CBAM, while the ResNet is the partial structure of CBAM-ResNet only without CBAM. Since the generation mechanisms of SMOTE and ADASYN are quite different from GAN-based methods and these methods cannot generate synchronizing signals, experiments based on a single sensor were also conducted. Experimental diagnosis results are detailed in Table 2, where the initial samples mean that the imbalanced samples are directly used for the training classifiers.

As shown in Table 2, the diagnosis accuracy based on the initial imbalanced samples declines obviously with the increase in imbalance degree. For example, the accuracy based on multiple sensors decreases from 97.38% to 94.3% when the imbalance ratio decreases from 0.5 to 0.1, while the accuracy based on a single sensor possesses a similar decrease tendency but with much lower accuracy from 94.83% to 89.8%. Besides, the decline rate with the increased imbalance degree of multiple sensors is much slower than that of the single sensor.

The accuracies significantly increase once the initial imbalanced data are expanded by different synthesis methods whether data from a single sensor or multiple sensors are adopted. It can be concluded that the imbalanced samples have a great influence on the classifier performance, and the data augmentation method can attenuate the imbalance influence. Among the generative methods, the proposed IDCGAN obtains the best diagnosis performance with any of the adopted classifiers. Taking the imbalance ratio of 0.1 and the ResNet classifier as examples, the proposed IDCGAN with multi-sensor can achieve a diagnostic accuracy of 98.49%, which is much higher than that of the initial sample of 94.3%. Moreover, this is also higher than the accuracy of the SN-DCGAN and WGANGP, which are 98.12% and 97.04%, respectively. Taking an imbalance ratio of 0.2 and IDCGAN as examples, the accuracy based on CBAM-ResNet is 99.54%, which is higher than the accuracy of CNN and ResNet with values of 98.52% and 98.61%. Furthermore, the proposed IDCGAN with the CBAM-ResNet method can achieve the best diagnostic accuracies in almost all imbalance ratios.

**Figure 11:**
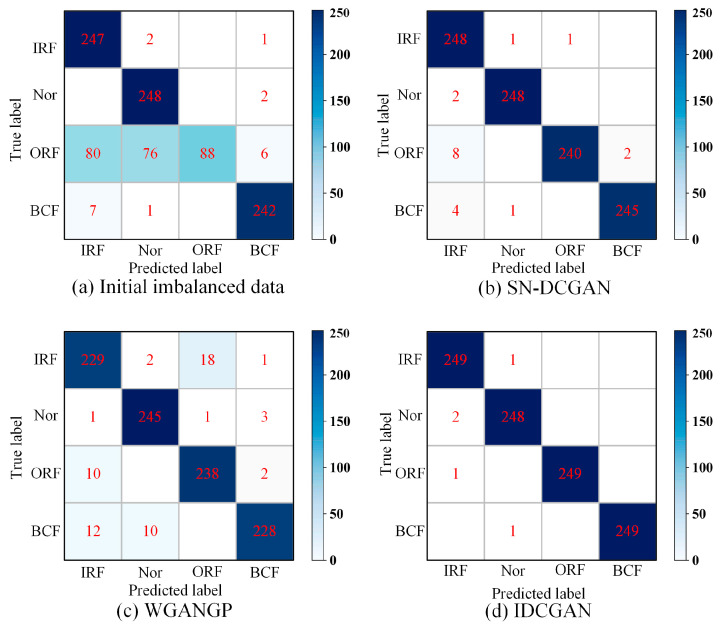
Confusion matrixes based on ResNet and different generation methods under limited ORF conditions with imbalance ratio of 0.1.

The confusion matrix is adopted to better illustrate the influence of different generative models on classification. Figure 11 shows the confusion matrixes based on the ResNet classifier under limited ORF conditions with an imbalance ratio of 0.1. It can be seen that a large number of ORF samples have been misclassified as other classes. For example, 80 and 76 ORF samples are misclassified as Nor and IRF while the classification of the majority class samples is correct. It indicates that the minority samples restrict the classifier from learning more valuable information. The misclassification of ORF is reduced when the synthetic samples generated by SN-DCGAN and WGANGP are adopted for data augmentation, and the misidentified samples are further reduced when the proposed IDCGAN is adopted.

In addition, single-class imbalanced fault diagnosis experiments based on IRF or BCF with an initial imbalance ratio of 0.2 are also performed. The results are shown in Table 3. The diagnosis results are consistent with the results based on ORF imbalanced data. For the single sensor, IDCGAN has higher diagnostic accuracy than other data synthesis methods, i.e., SMOTE and ADASYN whichever fault is limited. Moreover, the diagnosis accuracy of all experiments based on IDCGAN can reach above 98% while the initial accuracy with imbalanced data is less than 93.5%. As for multiple sensors, IDCGAN can also achieve the highest accuracy compared with other data synthesis methods, i.e., SN-DCGAN and WGANGP with the same classifier. In addition, the diagnosis accuracy-based CBAM-ResNet is the highest among the three classifiers. For example, the accuracy based on IDCGAN and CBAM-ResNet can reach 99.41% after the data augmentation in BCF limited occasion, which is 4.32% higher than that of CNN and 1.45% higher than that of ResNet. In conclusion, the proposed IDCGAN can well grasp the time-frequency sample distribution and generate high-quality samples for data augmentation. Moreover, the proposed CBAM-ResNet model can enhance the diagnosis accuracy and achieve satisfying results.

#### 4.1.5. Multi-Class Imbalanced Fault Diagnosis

**Figure 12:**
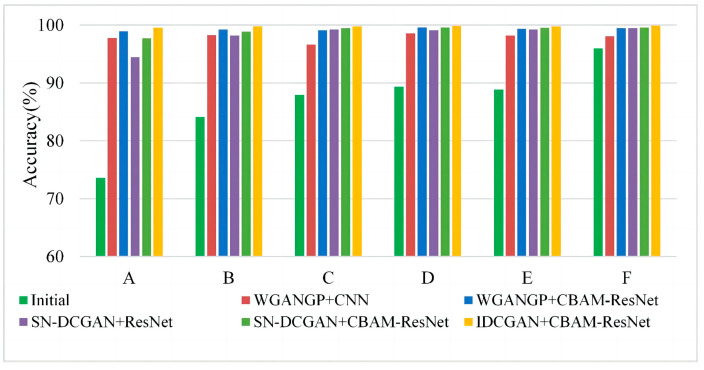
Experimental results based on multi-class imbalanced data.

In practice engineering, there always exist multiple fault imbalance problems. Thus, experiments based on multi-class imbalanced faults are performed to demonstrate the effectiveness of the proposed method. In this section, different imbalance ratios of multiple fault classes are selected as the initial training data to simulate multi-class imbalance occasions, which are detailed in Table 4. It should be mentioned that the majority of classes have 500 samples and the presented results are based on expanded balanced data except for the initial samples. Corresponding experimental results are shown in Figure 12. It can be seen that the accuracies of limited data with three fault classes (i.e., experiment A, B, and C in Table 4) are worse than that of only two limited fault classes data (i.e., experiments D, E and F in Table 4), especially when the initial samples are adopted for training classifiers. It is clear that the misclassification will decrease as the number of minority sample classes decreases. Furthermore, experiment results of all multi-class imbalanced fault diagnoses in Figure 12 indicate that the IDCGAN with CBAM-ResNet proposed in this paper can achieve the best diagnosis results, which is consistent with the results of single-class imbalanced experiments. The above results show that the proposed method also has excellent diagnosis performance on multi-class imbalanced datasets.

**Figure 13:**
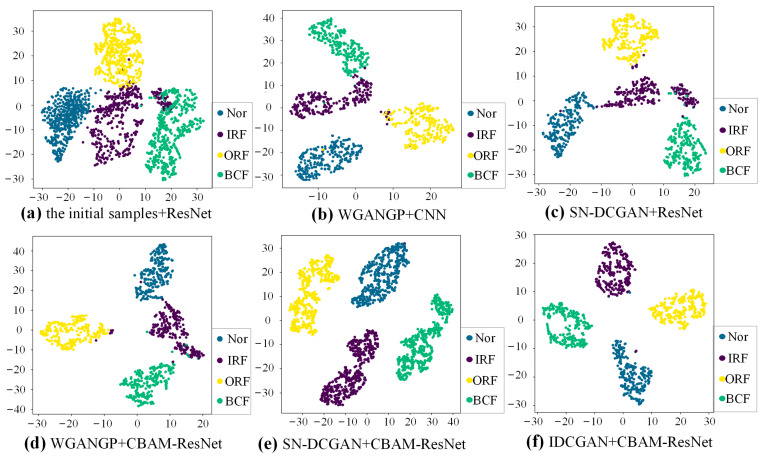
t-SNE visualization with different data augmentation methods and classifiers.

To better present the features of different generative methods and different classifiers, t-Distributed Stochastic Neighbor Embedding (t-SNE) is used as a qualitative method to evaluate the extracted high-dimensional sample feature. The t-SNE visualization results of extracted features before the fully connected layer of experiment A in Table 4 are shown in Figure 13. Each health state is represented by a special color. There exists apparent overlapping among the clusters of different health states with the initial samples, meaning that the classifier trained by imbalanced data cannot well identify samples of different health states. Comparing Figure 13a–c, it can be seen that the boundary becomes clearer once the classifier is trained with expanded balanced data. However, there still exist many overlaps when the CNN and the Resnet are adopted. The overlap has been improved by using the enhanced CBAM-ResNet, which is shown in Figure 13d,e. This also indicates the superiority of the proposed CBAM-ResNet. Compared with SN-DCGAN and WGANGP, the feature clusters are more compact when the IDCGAN is applied to the data augmentation. This indicates that self-attention can help to improve the quality of the generated samples which finally helps the classifier to better learn the deep features of the samples.

### 4.2. Case 2: Bearing Dataset from CWRU

#### 4.2.1. Dataset Description and Data Processing

The bearing dataset of CWRU is a kind of bearing dataset with artificial faults [35]. In this paper, the vibration signals collected under a load of 2 hp and with a sampling frequency of 12 kHz are adopted for the experimental validation. Moreover, signals measured by the acceleration sensors placed on the fan end (FE) and drive end (DE) are used as the basic multiple sensor data for the feature fusion. The same category fault with different dimensions is considered as one class, and finally, four health states are adopted, which are normal (Nor) state, inner race fault (IRF), outer race fault (ORF), and bearing roller fault (BRF).

The sliding window with overlap is adopted for the sample construction, whose window size and sliding step are set as 1280 and 480 points, respectively. Similar to Case 1, normalized synchronizing signal segments in the time domain from DE and FE are transformed to obtain the time-frequency feature with a size of 64 × 64 × 3 by CWT, which are then compressed and merged for the feature fusion. Finally, 750 time-frequency samples of each class are obtained. Moreover, 250 samples from each class are selected for model testing, and the remaining samples are randomly selected for the later generative model training according to experiment requirements.

#### 4.2.2. Evaluation of the Generated Samples

**Figure 14:**
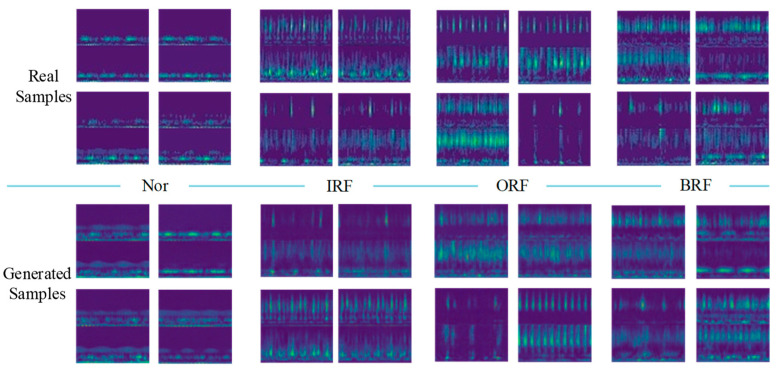
Comparison between the real samples and the generated samples with CWRU dataset.

Similar to the training process in Case 1, 100 samples of each class are used for training IDCGANs. Then, the well-trained GANs are applied to the sample generation. The visual comparison of the generated samples and corresponding real samples are shown in Figure 14. It can be seen that IDCGAN can generate high-quality samples that are remarkably similar to the corresponding real fused time-frequency samples, meaning that the IDCGANs have captured the distribution of real data and can generate high-quality synthetic samples with good similarity and some diversity.

The same strategy based on GAN-train is adopted to evaluate the performance of IDCGANs in this case. In this experiment, CBAM-ResNet is also adopted as the classifier for the fault diagnosis. As shown in Table 5, the diagnosis accuracy with the feature fusion method can reach 98.38%, which is 2.35% higher than that with a single sensor feature. Moreover, the standard deviation is much lower with the fusion time-frequency feature, showing the importance of the proposed fusion method. Besides, the GAN-train value with all generated samples from IDCGANs is about 97.58%, which is close to the GAN-base value of 98.38% and even a bit higher than the GAN-base with a single sensor. The accuracies and standard deviations of experiment B and experiment D are almost the same, which demonstrates the high similarity between the generated and real samples. In addition, seen from experiment E, the augmentation of generated samples can further increase the diagnostic accuracy to 99.17%, indicating that the generated samples also possess some diversity. In summary, IDCGAN is a reliable method that can be used for data generation and augmentation to improve the diagnostic performance of the classifier.

#### 4.2.3. Single-Class Imbalanced Fault Diagnosis

**Figure 15:**
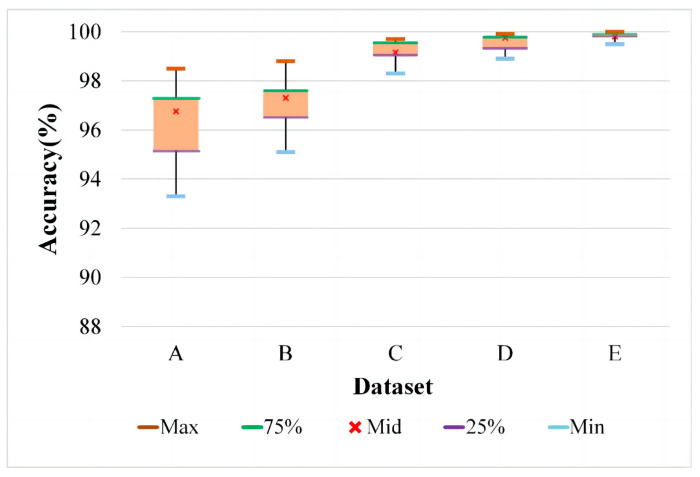
Experiment results with different numbers of generated samples.

In this section, BRF is selected as the minority fault class for the verification of imbalanced fault diagnosis. Fifty real samples of BRF are used for training the IDCGAN to generate new synthetic samples, which are gradually added to the initial data to alleviate the imbalanced degree; 500 real samples of each other class are randomly selected, which are combined with the augmented BRF samples to train a CBAM-ResNet for fault recognition. Finally, 500 synthetic samples generated by IDCGANs trained by 50 real samples of each class are added to further expand the balanced data to obtain better results. The components of data are shown in Table 6 and corresponding results are presented in Figure 15, in which the five markers in each box represent the maximum, upper quartile, median, lower quartile and a minimum of the 10 experimental results in each dataset. It can be observed that when the imbalance ratio of BRF is 0.1, the classifier cannot effectively learn the feature distinction among different health states, resulting in low accuracy and big fluctuations. With the increase in minority class samples, the diagnostic performance is significantly improved, and the accuracy is gradually stabilized. This shows that the samples generated by the data synthesis method IDCGAN are similar to the real samples.

Similar to the single-class imbalance experiment in Case 1, in this case, the IRF is chosen as the minority fault to study the effectiveness of the proposed method with various initial imbalanced data.

The initial imbalance ratios are set as 0.5, 0.2, and 0.1, which are directly expanded to balance data based on different generative methods except for the initial sample. As shown in Table 7, the diagnosis results based on a multi-sensor are better than those based on a single-sensor. For example, the diagnosis accuracy increased from 90.12% to 96.58% and 95.35% to 98.78% after multi-sensors are adopted when the initial imbalance ratio is 0.1 and 0.5, respectively. Moreover, the misclassification phenomena based on different classifiers are more obvious as the initial imbalanced degree increases. Furthermore, all the data synthesis methods can significantly improve the accuracy compared with the initial imbalanced samples. The proposed IDCGAN method provides the highest improvement among different generative methods based on the same classifier. Meanwhile, the CBAM-ResNet can reach higher diagnosis accuracy with the same data synthesis method and imbalance ratio. Therefore, the proposed IDCGAN + CBAM-ResNet together with the fused time-frequency feature is an effective way to enhance the diagnosis accuracy facing imbalanced data.

#### 4.2.4. Multi-Class Imbalanced Fault Diagnosis

**Figure 16:**
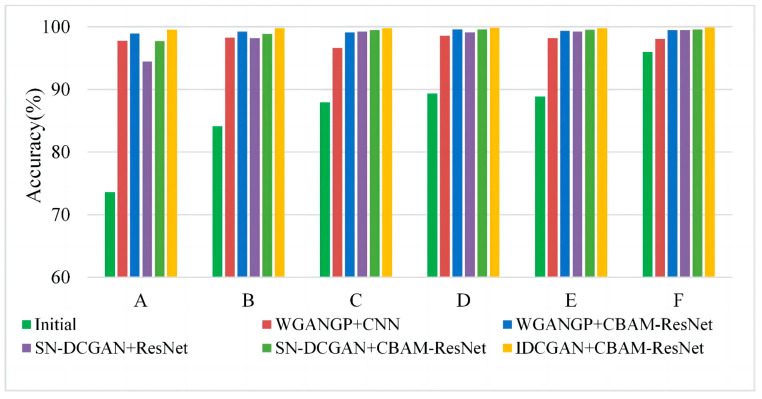
Experimental results based on multi-class imbalanced data.

Similar to Case 1, the multi-class imbalanced fault diagnosis including the two classes and three classes of fault imbalance experiments are performed. For multi-class imbalanced experiments in this case, the BCF in case 1 is replaced by BRF while the other setups are the same. The comparison results are shown in Figure 16. It can be seen that if the initial samples are imbalanced and without an augmentation, the classifier will not be able to learn deep features effectively, resulting in misclassification with the decrease in the minority class samples. In each dataset, after the samples are balanced by these data synthesis methods, the diagnostic performance will be improved accordingly, and the IDCGAN-CBAM-ResNet can achieve better diagnostic results than other methods.

**Figure 17:**
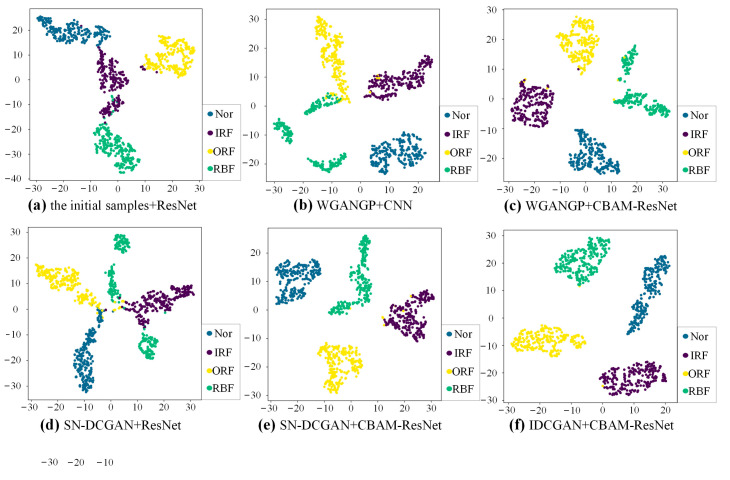
The t-SNE visualization results with different methods.

The t-SNE visualization results of high-dimensional features learned by different classifiers with different augmented training data are shown in Figure 17. The results are obtained based on the same experiment as case 1. It is clear that the feature clusters between different samples cannot be well distinguished with initial imbalanced data. The feature cluster boundaries of different classes based on CBAM-ResNet and expanded balance data are much clear than those of the CNN and ResNet, indicating the strong feature extraction ability of the proposed CBAM-ResNet. Besides, the feature clusters become compact and there is almost no overlap, meaning that the proposed IDCGAN has a strong sample generation ability and is superior to the generative methods used for comparisons.

From the above, it can be concluded that the fused time-frequency feature based on the multi-signal fusion method is beneficial to provide more valuable information. The proposed IDCGAN can generate samples that have a similar distribution to real samples, which is better than the comparison methods. Moreover, the CBAM-ResNet can better distinguish the fused time-frequency feature of different faults than the comparison classifiers. Therefore, the proposed method can address the imbalanced data problem and enhance the diagnosis accuracy.

## 5. Conclusions

In order to reduce the unfavorable impact of imbalanced data in intelligence fault diagnosis and improve fault diagnosis accuracy, a novel bearing fault diagnosis method is proposed. Firstly, signals from different sensors are transformed into time-frequency images by CWT, which are then compressed and spliced to fuse the features of multiple sensor signals. Subsequently, the self-attention mechanism, SN, and Wasserstein distance with GP are introduced to construct the IDCGAN model to ensure a stable training process and improve the generated sample quality. Finally, the CBAM is introduced into the residual network to extract deep features of the augmented balance dataset for better fault diagnosis results. Experiments based on two different bearing datasets, i.e., the life-cycle bearing fault and manufactured bearing fault, have demonstrated that the proposed IDCGAN + CBAM-ResNet method together with the fused time-frequency feature is beneficial to generate high-quality samples and can achieve better diagnostic performance than the comparison methods, showing great potential in imbalanced fault diagnosis. However, the proposed IDCGAN is not applicable once the training sample is extremely scarce and cannot be applied to generate fault samples with unknown features. Therefore, corresponding research will be carried out to meet the challenges of data generation with 1 shot real sample and data generation of related but unknown features in the future.

## Figures and Tables

**Table 1:** Experimental setup and results for evaluation of the generated samples.

Experiments	Signal Source	Sample Component			Accuracy (%)	Standard Deviation (%)
		Real	Generated	Total		
A	Single senso	100	0	100	89.78	2.51
B	Multi-sensor	100	0	100	96.89	1.21
C		0	100	100	92.35	1.82
D		50	50	100	96.23	0.65
E		100	100	200	98.83	0.61

**Table 2:** Results after data augmentation of ORF with different initial imbalance ratios.

Source	Methods	Classifier	Accuracy with Different Imbalance Ratios (%)		
			0.1	0.2	0.5
Single sensor	Initial sample	ResNet	89.8	92.32	94.83
	SMOTE	ResNet	95.25	96.86	97.88
	ADASYN	ResNet	95.78	97.22	98.56
	IDCGAN	ResNet	98.13	98.53	98.7
Multi-sensor	Initial sample	ResNet	94.3	95.02	97.38
	SN-DCGAN	CNN	97.33	97.49	97.73
		ResNet	98.12	98.17	98.57
		CBAM-ResNet	99.14	99.26	99.39
	WGANGP	CNN	95.48	98.34	98.47
		ResNet	97.04	97.54	98.3
		CBAM-ResNet	99.42	99.4	99.34
	IDCGAN	CNN	98.53	98.52	98.65
		ResNet	98.49	98.61	98.8
		CBAM-ResNet	99.5	99.54	99.64

**Table 3:** Diagnosis results of different minority faults with imbalance ratio of 0.2.

Source	Methods	Classifier	Accuracy (%)		
			IRF	ORF	BCF
Single sensor	Initial samples	ResNet	93.3	92.32	90.89
	SMOTE	ResNet	96.85	96.86	96.73
	ADASYN	ResNet	97.57	97.22	96.74
	IDCGAN	ResNet	98.72	98.53	98.15
Multi-sensor	Initial samples	ResNet	94.15	95.02	91.31
	SN-DCGAN	CNN	97.07	97.49	95.3
		ResNet	98.23	98.17	97.67
		CBAM-ResNet	99.05	99.26	99.11
	WGANGP	CNN	98.78	98.34	93.74
		ResNet	95.73	97.54	96.14
		CBAM-ResNet	98.93	99.4	99.29
	IDCGAN	CNN	99.16	98.52	95.09
		ResNet	98.93	98.61	97.96
		CBAM-ResNet	99.29	99.54	99.41

**Table 4:** Experiment description of multi-class imbalanced data.

Experiment	Sample Description
A	BCF 0.1, IRF 0.1, ORF 0.1, Nor 1
B	BCF 0.1, IRF 0.2, ORF 0.2, Nor 1
C	BCF 0.1, IRF 0.2, ORF 0.5, Nor 1
D	BCF 0.2, ORF 0.2, IRF 1, Nor 1
E	BCF 0.1, IRF 0.2, ORF 1, Nor 1
F	BCF 1, IRF 0.5, ORF 0.2, Nor 1

**Table 5:** Experimental setup and results for evaluation of the generated samples.

Experiments	Signal Source	Sample Component			Accuracy (%)	Standard Deviation (%)
		Real	Generated	Total		
A	Single-sensor	100	0	100	96.03	2.45
B	Multi-sensor	100	0	100	98.38	0.55
C		0	100	100	97.58	1.82
D		50	50	100	98.35	0.57
E		100	100	200	99.17	0.37

**Table 6:** Experiment components with different amounts of generated samples.

Dataset	Sample Ratio	Minority Class (BRF)			Other Classes		
		Real	Generated	Total	Real	Generated	Total
A	1:10	50	0	50	500	0	500
B	1:5	50	50	100	500	0	500
C	1:2	50	200	250	500	0	500
D	1:1	50	450	500	500	0	500
E	2:2	50	950	1000	500	500	1000

**Table 7:** Results after data augmentation of IRF with different initial imbalance ratios.

Source	Methods	Classifier	Accuracy with Different Imbalance Ratios (%)		
			0.1	0.2	0.5
Single sensor	Initial sample	ResNet	90.12	93.23	95.35
	SMOTE	ResNet	96.47	97.56	98.04
	ADASYN	ResNet	97.23	97.9	98.49
	IDCGAN	ResNet	97.95	98.55	98.9
Multi-sensor	Initial sample	ResNet	96.58	97.75	98.78
	SN-DCGAN	CNN	97.9	98.37	98.84
		ResNet	98.82	98.98	99.26
		CBAM-ResNet	99.03	99.19	99.53
	WGANGP	CNN	96.99	98.14	98.69
		ResNet	97.21	98.88	99.07
		CBAM-ResNet	99.4	99.48	99.65
	IDCGAN	CNN	98.15	99.08	99.33
		ResNet	99.34	99.53	99.45
		CBAM-ResNet	99.74	99.82	99.89

## Data Availability

Not applicable.
